# Sexual selection and reproductive success in mesocosm populations of African annual killifish

**DOI:** 10.1111/jfb.70433

**Published:** 2026-03-29

**Authors:** Jakub Žák, Klára Mrkvová, Martin Reichard

**Affiliations:** ^1^ Department of Botany and Zoology, Faculty of Science Masaryk University Brno Czech Republic; ^2^ Institute of Vertebrate Biology Czech Academy of Sciences Brno Czech Republic; ^3^ Department of Ecology and Vertebrate Zoology University of Lodz Lodz Poland

**Keywords:** mate choice, *Nothobranchius furzeri*, seasonal fish, territoriality

## Abstract

Variation in individuals’ ability to obtain mates generates sexual selection, which typically acts more strongly on males and can produce pronounced differences in phenotypes between the sexes (i.e., sexual dimorphism). The dynamic of sexual selection and individual reproductive success is considerably affected by the availability of reproductive territories. *Nothobranchius furzeri* Jubb, 1971 (a model system in aging studies) inhabits temporally and spatially constrained ephemeral savanna pools where it lays its eggs in the muddy substrate. Males of the species are large and aggressive; nonetheless, it is unclear why it is dimorphic, whether they form territories and how females select their partners for mating. We released 96 individuals into 12 semi‐natural mesocosms with either a clustered or a dispersed configuration of spawning substrate. We found that males formed body size‐dependent dominance hierarchies. Dominant males were territorial and nearly monopolized matings when the substrate was defendable. Females engaged in 40% more spawning acts within a spawning bout with dominant males. Therefore, the intersexual size dimorphism appears to be maintained by the higher competitive ability and reproductive success of large males. Females were less aggressive, but were harassed by males, and fed three times more than males. Overall, we demonstrated under a semi‐natural setting that the mating system in *N. furzeri* is dominated by large males, which become territorial when the spawning substrate is limited (clustered), a likely situation in the environment of ephemeral pools.

## 1 INTRODUCTION

Individual differences in reproductive success generate sexual selection, which typically acts more strongly on males and can produce pronounced differences between the sexes, such as sexual dimorphism in phenotypic traits or differences in behavioural repertoires (Clutton‐Brock, 2017; Magurran & Maciás Garcia, 2000). Large body size improves male competitive ability (Kodric‐Brown, 1988; Konečná et al., 2010) and often leads to the formation of a size‐related hierarchy, where the dominant male secures most matings (Clutton‐Brock & McAuliffe, 2009). Although dominance greatly contributes to reproductive success in territorial species with limited breeding resources, male territoriality may not pay off when these resources are abundant or undefendable (Konečná et al., 2010).

Female choice is limited in social systems with high potential for males to monopolize breeding resources, and with coercive mating (Clutton‐Brock & McAuliffe, 2009; Clutton‐Brock & Parker, 1995; McCann, 1981; Muller et al., 2011). Yet, females use counter‐strategies to reduce their reproductive costs and may reduce oviposition when mating with a non‐preferred partner or on a suboptimal spawning substrate (Bacon & Barbosa, 2020; Reyer et al., 1999; Smith et al., 2000; Yamazaki & Koizumi, 2017). The act of mating coercion is itself costly to females (Trivers, 1972), and the cost further broadens through the interference with the female foraging (Magurran & Seghers, 1994). In continuous breeders, foraging time and ration are important correlates of fecundity (Vrtílek & Reichard, 2015) and females spent a substantial amount of time on feeding. Therefore, any interference with foraging directly decreases female reproductive success.

African seasonal killifish from the genus *Nothobranchius* are extremely dimorphic, with colourful, large males and drab, smaller females (Reichard & Polačik, 2018). *Nothobranchius furzeri* Jubb, 1971 is a small (males: 5–7 cm, females: 3–5 cm) fish species living only for several weeks to months in isolated, ephemeral savanna pools in southeastern Africa (Reichard & Polačik, 2018). They reproduce daily, with females laying dozens of eggs (Vrtílek & Reichard, 2016), suggesting strong selection on female fecundity. Male‐biased body size dimorphism in this species indicates a possible role of male dominance on reproductive success.

Despite the wide use of *N. furzeri* in studies spanning from life‐history evolution to evolutionary genomics (Bartáková et al., 2013; Vrtílek, Žák, Pšenička, & Reichard, 2018; Willemsen et al., 2020), the role of sexual selection in *N. furzeri* reproductive success has been little studied. Males are aggressive, and it is assumed that they may defend spawning territories (Cellerino et al., 2016; Reichard et al., 2009), compete for access to females and form dominance hierarchies (Cellerino et al., 2016). However, any empirical study on their mating system is missing.

Therefore, the aims of the present study were to: (1) determine the role of body size in male dominance; (2) examine whether dominant males achieve higher reproductive success when the spawning substrate is a limiting and defendable breeding resource; (3) test whether females adjust their oviposition in relation to male and substrate traits; (4) evaluate how the sexes differ in their behaviour and (5) compare male territoriality under dispersed and clustered spawning substrate configurations. Overall, this study aimed to clarify the causes and consequences of sexual dimorphism in *N. furzeri* and estimate the role of sexual selection in the temporally and spatially constrained conditions of ephemeral savanna pools.

## 2 MATERIALS AND METHODS

### 2.1 Experimental fish and set‐up

Ninety‐six adult, 3‐month‐old *Nothobranchius furzeri*, non‐inbred captive strain MZCS 024 (Cellerino et al., 2016) were housed in 12 outdoor fibreglass tubs (bottom dimensions 1.2 × 1.2 m) filled to a depth of 25 cm, with clear water in the garden of the Institute of Vertebrate Biology (IVB), Brno, Czechia. Each tub contained eight randomly selected fish in a 1:1 sex ratio [body size range (TL): male = 42–64 mm, female = 33–46 mm; fish density 5.6 fish per m^2^], which is within an ecologically relevant density of 0.22–40 ind. per m^2^ (Vrtílek, Žák, Polačik, et al., 2018). Fish were habituated to the mesocosms for 1 week before the experiment. Seven days before the habituation period, each fish was marked subcutaneously by coloured elastomers (VIE – visible implant elastomer, Northwest Marine Technology Inc., USA) into the dorsal part of the body to enable individual recognition during behavioural observations. Fish fed on natural biota, which fell and/or established in tubs, and were further supplemented once per day between 8:00 a.m. and 10:00 a.m. by frozen bloodworms (Chironomidae larvae) in the amount consumed within 3 min. The behavioural scoring was performed at least 1 h after feeding to avoid the immediate effect of feeding activity on fish interactions. After the experiment was completed, the fish were euthanized and their body size measured.

### 2.2 Spawning substrate and environmental variables

We tested the role of two substrate configurations on fish behavioural interactions. The spawning substrate was fine silicate sand (0.4–4 mm; Polačik et al., 2016) placed in four plastic containers (11 × 8 × 6 cm) as a 3 cm deep layer. Half of the mesocosms had a clustered configuration of spawning substrate, where all four plastic containers were placed in one corner. In the other six mesocosms, the configuration of the spawning substrate was dispersed, with one container placed in each corner of the tub. The bottom of each tub was divided into four equally sized quadrants to record the position of fish within the tub during behavioural observations. Fish were exposed to the natural temperature and photoperiod during the Central European temperate summer between 24 July and 9 August 2019. Water temperature was monitored in each tub using a HOBO Pendant Temperature 64 K Data Logger, USA. Temperature ranged between 19.1 and 33.5°C, which is within the natural temperature range experienced by wild fish (Reichard, 2016; Žák et al., 2018). The behaviour was scored during the daylight (from 7:00 a.m. to 8:30 p.m.). This corresponds to the period of activity in wild populations (Žák et al., 2019).

### 2.3 Scoring behaviour

**FIGURE 1:**
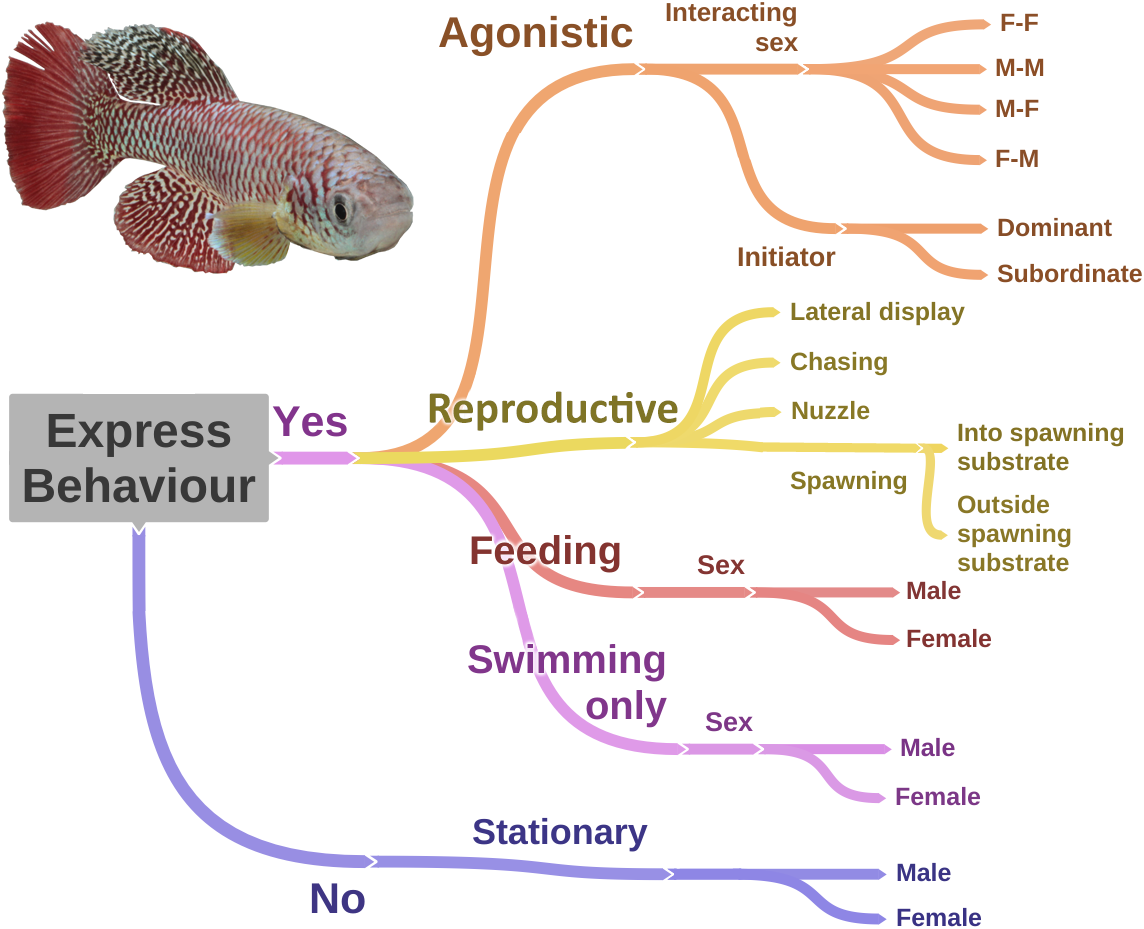
The flowchart of recorded behaviours in the mesocosm experiment with turquoise killifish (*Nothobranchius furzeri*). Within agonistic behaviour, the interacting sex subcategories are represented on the left by the initiator of the conflict and the recipient on the right (F – female, M – male). In the initiator of agonistic behaviour, dominant is meant as the initiator and subordinate is the recipient of the agonistic behaviour. Agonistic behaviours included chasing, lateral display, opercular flaring, biting, mouth‐wrestling and their combinations. Specific types of agonistic behaviours were not recorded because they were usually performed as combinations and some (opercular display, lateral display) were difficult to recognize by observing fish from above. For the type of reproductive behaviour, only the most advanced form which occurred during the observation period was recorded. This means that spawning typically involves also chasing and nuzzle, but only the spawning was recorded. Typically, males were initiators of the epigamic behaviour when they initially chased a female and displayed to her during the stops when they performed lateral displays (difficult to observe from above). The second phase is nuzzle when the male is in the dorso‐ventral (male above female) position and leads female towards the spawning substrate. They spawn as a pair when the male enfolds the female by the dorsal and anal fins, and both are performing jerking movements during which they release gametes. ‘Swimming only’ was recorded when fish was only swimming without any interaction with other fish and swimming was impossible to associate with another behaviour. ‘Stationary’ was recorded when an individual was motionless during an observation interval. All behaviours are within the repertoire of both sexes. Each record was paired with the time of the day and the water temperature in the tub. Recording according to this flowchart was not used for the additional observations focused solely on spawning.

Five categories of behaviour were scored. They included: (1) agonistic behaviour, (2) reproductive behaviour, (3) feeding behaviour, (4) swimming only and (5) stationary position (see description in Figure 1). These behaviours were partially adapted from the work of Haas (1976) on *Nothobranchius guentheri* and adjusted to *N. furzeri* in accordance with our long‐term breeding experience of this species (Cellerino et al., 2016). All behavioural records (not video‐recorded) were completed by a single observer (K. M.), and observer consistency was validated by J. Z. during seven simultaneous observation sessions. For each observation, one randomly chosen individual was observed for 1 min (measured by a stopwatch), and only the behaviours observed for this individual were recorded (not for other fish in the tub, even if they were directly interacting with the observed individual). After 1 min, all observed behaviours of a focal individual were recorded in the protocol as outlined in the flowchart in Figure 1. The number of units for each behaviour was recorded (except for swimming and stationary, which were recorded as presence/absence data). Each record contained the time of the day of the observation. For analysis, we pooled data from 7:00 a.m. to 2:00 p.m. as the first half‐day (morning) period and 2:00 p.m. to 8:30 p.m. as the second half‐day (afternoon) period to account for our analyses for behavioural periodicity. Behavioural data were linked to water temperature data (recorded by a datalogger) for the analysis to control for the effect of temperature on fish behaviour.

In total, fish were observed  (not consecutive) 11 days for at least 12 min per day, resulting in 768 one‐minute observation intervals during which 1,895 behaviours (including stationary) were recorded. The number of recorded behaviours for an individual fish varied from 7 to 49 (1–14 records for a single interval). Both treatments and sexes were observed with equal frequency.

### 2.4 Spawning data

Male and female reproductive success was determined from the additional data collection focused solely on spawning behaviour, because spawning was recorded rarely during standard observations (only 69 spawning observations representing 3.6% of all records). The IDs of both individuals of the interacting pairs were recorded. Additional data collection yielded 111 observation intervals of spawning behaviour. Each tub was observed directly by K. M. for 5 min, and whether each individual within the tub spawned or not (1/0), and the number of spawning acts within each spawning bout was recorded. Separate spawning acts were scored when a pair persisted in the clasping position, rested for 1–2 s between consecutive spawning acts (i.e., jerking movements of male and female tails; see illustrative Video S1). When fish were disturbed during observation by wind, birds or the observer (16 cases), only the observations lasting longer than 3 min were included (six cases <3 min were removed). The location of spawning (i.e., inside or outside of the spawning substrate) was always recorded. The observation time for the additional spawning dataset was targeted at the second half‐day, when spawning activity within the mesocosm (from the previous dataset) reached the highest rate (see Figure S1). Because spawning observations were completed during a short time window (a few hours of the afternoon), time of the day was not included in the spawning analysis as a predictor.

### 2.5 Statistical analysis

Predictors of *male dominance* (ratio between initiated and received aggression towards/from conspecifics) were determined by a generalized linear mixed effect model (GLMM) with binomial error distribution. Predictors were *male body mass* (continuous), *substrate configuration* (two levels: clustered, dispersed), *water temperature* (continuous) and *time‐of‐the‐day* (two levels: morning, afternoon). *Male ID* (48 levels) nested within *Tub ID* (12 levels) were nested random effects, and *observation ID* (383 levels) was a simple random effect.

The spawning probability of males [whether spawning was observed over the 5 min observation interval (1/0)] in relationship to male *dominance* (four levels, dominance order determined from the initial dataset), *body mass* (*bm*) and *substrate configuration* was tested by binomial GLMM using the additional spawning dataset. *Male ID* was a nested random factor within *Tub ID*, and ID of observation interval (91 levels) was a simple random factor. The same model structure was used for assessing female spawning probability. Significant interactions of *dominance:substrate* and *bm:substrate* were retained in the final model for males.

To test which male traits affect their probability of spawning into the spawning substrate, a GLMM with a binomial error distribution was used. The proportion of the individual sums of spawnings per minute into the substrate/outside the substrate (summed over all observation intervals for each male) was a response variable, and *male dominance* (two levels, dominant vs. all subordinate), *male body mass* and *substrate configuration* were predictors. *Tub ID* was treated as a random factor. This was completed only for 39 males, which were observed spawning during the ‘additional spawning’ observation.

Male territoriality was analysed as the relative frequency of male presence in a specific quadrant by a baseline categorial multinomial model (BCMM) (Elff, 2022) for tubs with clustered substrate only, because it was possible to distinguish the quadrant with a spawning substrate from other quadrants. *Quadrant* (four levels: with substrate, diagonal to substrate, left to substrate, right to substrate) was a response variable and *dominance* category of male (four levels), *time‐of‐day* and *water temperature* were predictors. Random effects were *Fish ID* nested within *Tub ID*, and the simple random effect of *observation ID* (189 levels). In the case of tubs with dispersed spawning substrate position, only tub‐specific proportions of quadrant visits by males were analysed (Table S1).

To test whether male territoriality is affected by an increased presence of females around the spawning substrate in the clustered treatment, female preference for specific quadrants was tested by BCMM with *quadrant* as a response variable and *time‐of‐day* and *water temperature* as predictors. Tub ID (six levels) with nested female ID (24 levels) were random factors, and observation interval (189 levels) was a random factor.

Female choice in terms of the duration of spawning (i.e., number of spawning acts) per spawning bout (*N* = 185) in relation to male *dominance* (two levels) and *substrate preference* (two levels, inside/outside substrate) was tested by Gamma GLMM. Also, *substrate configuration in the tub* and female *body mass* were involved as model predictors. *Female ID* (41, no records for 7 females) nested within *Tub ID* was a nested random factor, and *ID of observation interval* (72 levels) was a simple random factor. Within treatment (substrate configuration), marginal means were estimated by the *emmeans* package version 1.8.5. (Lenth, 2023).

To compare sex‐specific behavioural profiles, BCMM was used. The model contained a response variable of observed behaviours (five levels, Figure 1) and predictors: *sex* (two levels), *substrate configuration*, *time‐of‐day* and *water temperature*. Random effects were *Fish ID* (96 levels) nested within *Tub ID* (12 levels), and the *observation interval* (768 levels), which was treated as a simple (non‐nested) random effect. The most parsimonious model contained significant interactions of *sex:time‐of‐day*, *sex:substrate configuration* and *time‐of‐day:temperature*. Interactions involving the factor of *sex* appeared to have a small biological effect despite being statistically significant, and we were primarily interested in sex‐specific behavioural profiles; therefore, we briefly report the results of interactions in Figure S1. Sex‐specific marginal means were generated by *emmeans* package version 1.8.5. (Lenth, 2023) from the model, including all significant interactions. To test whether a very high proportion of male aggression was a sole driver of sex‐specific differences, the model of behavioural types was fitted again without agonistic behaviour, which provided qualitatively comparable outcomes (not shown).

The dynamic of each type of reproductive behaviour (three levels, Figure 1) and direction of agonistic interactions (three levels) in relation to *time‐of‐day* and *substrate configuration* was tested by BCMM applying the same model structure and approach as in the paragraph above (except repeating analysis without agonistic interactions).

All models were fitted with all possible two‐way interactions, which were removed by backward selection one at a time when insignificant. The predictor significance of BCMM models was estimated by Analysis of Deviance comparison of models with and without the predictor/interaction of interest, and therefore, only *p*‐values could be reported. All models were checked for collinearity by the variance inflation factor (VIF) from the *car* package version 3.1.2 (Fox & Weisberg, 2019), and all predictors were below VIF of 2.5. All BCMM models, as well as GLMM models with binomial distribution, were checked for overdispersion (commands check_overdispersion, dispersion; Elff, 2022; Lüdecke et al., 2021), and none deviated (reaching values 0.7–1.3). All post hoc pairwise contrasts of marginal means were produced by *emmeans* (Lenth, 2023) if not specified differently. Statistical analysis was done in the R environment version 4.2.2 (R Core Team, 2024). A summary of the statistical approaches is provided in Table S2.

## 3 RESULTS

### 3.1 Male dominance, spawning frequency and substrate preference for spawning

**FIGURE 2:**
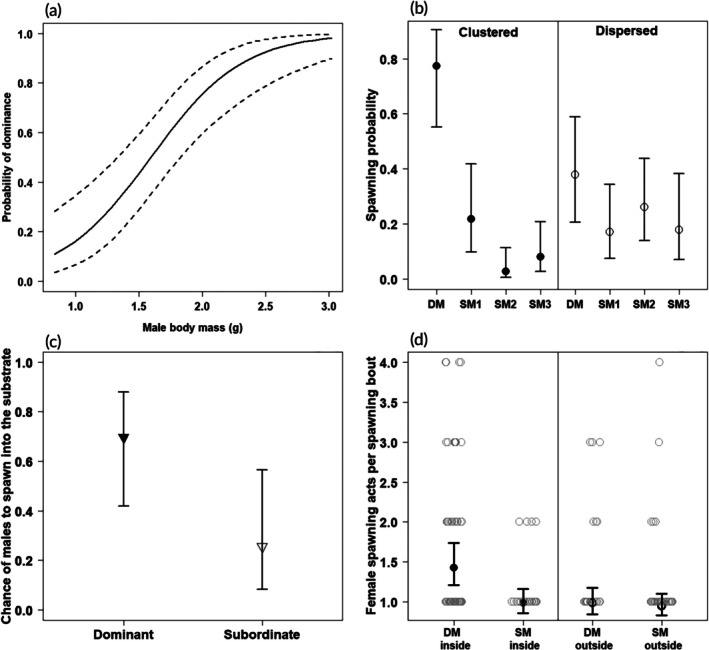
Male‐dominance‐related traits and reproductive behaviour in mesocosm populations of *Nothobranchius furzeri*. (a) Relationship between dominance and body size in male *N. furzeri* (*N* = 631). Male dominance was positively related to body mass. Male dominance was determined as a ratio of initiated to received agonistic interactions. The dotted line represents the 95% confidence interval (CI) from the binomial generalized linear mixed effect model (GLMM). (b) The probability of observing male spawning in relation to its dominance and spawning substrate configuration (*N* = 91). The dominant males spawned proportionally more frequently when the substrate was clustered, which is in contrast to treatments with dispersed spawning substrate, where all males had a similar probability of spawning. Y‐axis is on relative scale (0–1) of how spawnings were distributed among males. Points are model‐estimated means from Bin GLMM. DM – dominant male, SM1‐3 – subordinate males. (c) Higher probability of dominant males to spawn into the spawning substrate (*N* = 39). All subordinate males were pooled because they did not statistically differ from each other. Points are means from Binomial GLMM. (d) Mean number of female spawning acts during the single spawning bout in relation to male dominance and position of spawning (inside/outside the spawning substrate). Females spawned more often with dominant males when they were spawning into the spawning substrate than with subordinate males. Black points are estimates from Gamma GLMM. Grey circles are points of observation, jittered on *x*‐axis for better visibility. Points of observation are shown only for non‐binary data. Plotting observation points for binary data was difficult to read due to large sample sizes. Error bars in all plots are 95% CI (*N* = 185).

Male dominance was positively related only to body mass (Binomial GLMM, χ^2^_1_ = 19.88, *p* < 0.001; Figure 2a, Table S3). The most dominant males nearly completely monopolized spawning when the substrate was clustered (Binomial GLMM, *dominance:substrate* interaction, χ^2^_3_ = 12.74, *p* < 0.001; Table S4, Figure 2b). In contrast, when the substrate was dispersed, spawning rate only depended on body mass but not on dominance (*body mass:substrate*, χ^2^_3_ = 4.45, *p* = 0.035; Figures 2b and S2). Dominant males spawned twice more frequently into the spawning substrate than subordinate males (Binomial GLMM, χ^2^_1_ = 4.23, *p* = 0.039; Figure 2c, Table S5), irrespective of substrate configuration (χ^2^_1_ = 0.07, *p* = 0.794) or male body mass (χ^2^_1_ = 0.13, *p* = 0.720).

### 3.2 Territoriality

Dominant males formed territories near clustered substrate, as 59% (±7%) of their recorded positions were located in the quadrant containing substrate. This was twice as much as the rest of the males in a tub (estimated pairwise marginal means from BCMM, means for subordinate males: 20%–28%, se = 8%, z = 3.91–5.20, all *p* < 0.001; Table S1). In addition, subordinate males were found more frequently in the most distant quadrant (diagonal) to the quadrant with a spawning substrate (means for subordinate males = 23%–29%) than the dominant male (6%) (se = 5%, z = 3.10–4.14, *p* = 0.001–0.011; other statistics in Table S6). There was no preference for a specific quadrant for the dominant male in the dispersed substrate treatment (Table S1). Male territoriality was not affected by female distribution in the clustered substrate treatment as females moved freely among quadrants (estimated pairwise marginal means from BCMM, z = −1.49–1.71, *p* = 0.262–0.995).

### 3.3 The direction of agonistic behaviour

**FIGURE 3:**
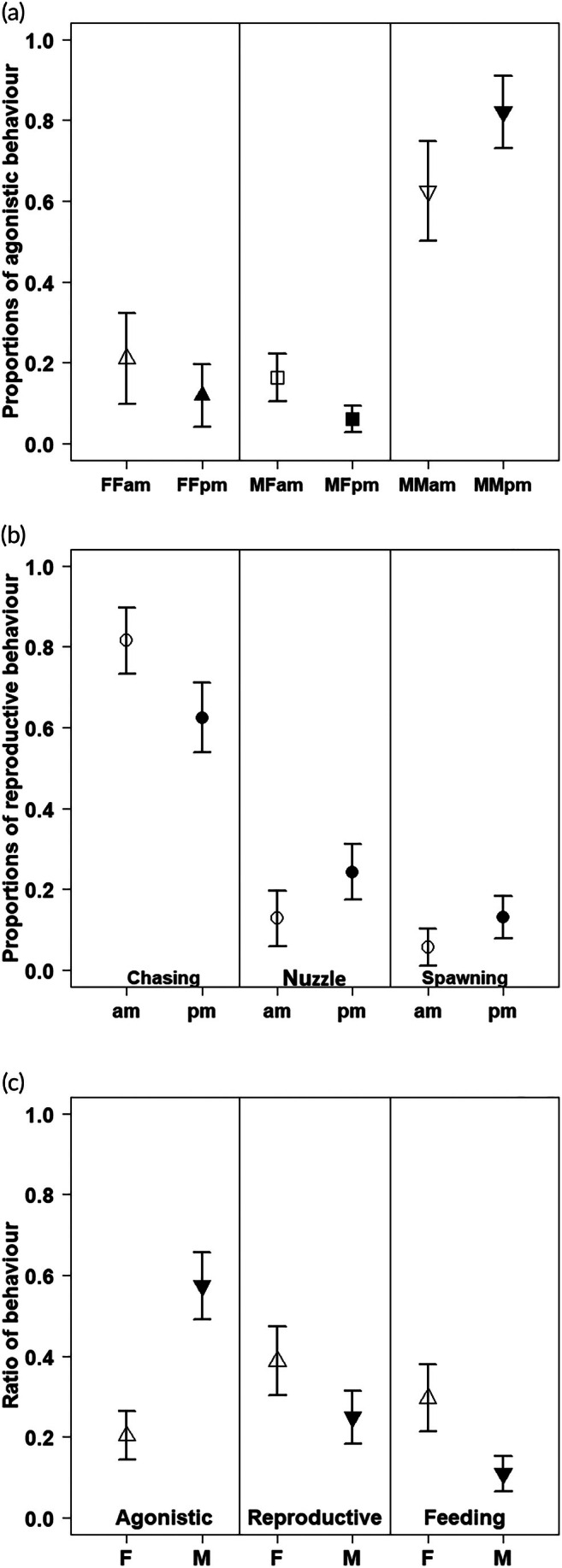
Structure of the sex‐specific behavioural repertoire, direction of agonistic behaviour and structure of reproductive behaviour. (a) Direction of agonistic behaviour in relation to time of day (*N* = 800). FF – female aggressive towards female, MF – male aggressive towards female, MM – male aggressive towards male. a.m. – morning, p.m. – afternoon. (b) Time‐of‐day‐dependent structure of reproductive behaviour (*N* = 530). a.m. – morning, p.m. – afternoon. (c) Sex‐specific behavioural profile of the mesocosm populations of Nothobranchius furzeri. F – female, M – male. Detailed relationships, including daytime effects and behaviours such as swimming‐only and stationary behaviour, are provided in Figure S1. Points are baseline categorial multinomial model (BCMM) estimated means, and error bars are 95% CI.

The majority of aggression was intrasexual (males: 73%, *N* = 584/800, females: 16%). Intersexual aggression was rare, with more male aggression towards females (10%) and nearly no female aggression towards males (1%, *N* = 8/800, omitted from further analyses). Male–male aggression was 20% more frequent in the afternoon than in the morning (estimated pairwise marginal means from BCMM, z = 3.66, *p* < 0.001; Figure 3a). Male–female aggression (z = 2.96, *p* = 0.003; Figure 3a) as well as female–female aggression (z = 2.25, *p* = 0.026; Figure 3a) were twice as prevalent in the morning as in the afternoon. Neither substrate configuration (*p* = 0.628) nor temperature (*p* = 0.762) affected the direction of agonistic behaviour.

### 3.4 The structure of reproductive behaviour

The most frequently observed reproductive behaviour was chasing (63%, *N* = 340/537), followed by nuzzling (23%) and spawning (13%). Lateral display was observed rarely (1%, *N* = 7/537) and omitted from further analyses. Chasing was 19% more frequently observed in the morning than in the afternoon (estimated pairwise marginal means from BCMM, z = 3.85, *p* < 0.001; Figure 3b). In contrast, more downstream reproductive behaviours were more prevalent in the afternoon (z = 2.38–2.70, *p* = 0.007–0.018; Figure 3b). The occurrence of specific types of reproductive behaviours was not dependent on substrate configuration (*p* = 0.387) or temperature (*p* = 0.424).

### 3.5 Female mate choice

Female spawning probability was not significantly related to any predictor (Table S7). When females spawned into the spawning substrate, they completed 40% more spawning acts with dominant males, irrespective of treatment (Gamma GLMM, *male dominance: place of spawning* interaction, χ^2^_1_ = 8.00, *p* = 0.004; Figure 2d, Table S8).

### 3.6 Sex‐specific behaviour

Contrasting reproductive roles of each sex were expressed as sex‐specific behavioural profiles. Males were 2.5 times more frequently involved in agonistic behaviour than females (estimated pairwise marginal means from BCMM, z = 11.23, *p* < 0.001; Figure 3c). Females were eating three times more frequently than males (z = 6.26, *p* < 0.001; Figure 3c). Females were involved in reproductive behaviour 10% more frequently than males (z = 3.67, *p* = 0.002; Figure 3c). However, the number of per‐minute reproductive behaviours was similar between sexes (Table S9). The summary of sex‐specific behavioural profiles (activity budgets) and the frequency of each behaviour per 1 min are detailed in Tables S9 and S10.

Sex‐specific behavioural profiles diverged throughout the day (BCMM, *sex:time‐of‐day* interaction, *p* < 0.001) with decreasing female feeding activity and increasing male agonistic interactions (Figures 3a, S1). Substrate configuration had a weak effect on the sex‐specific behavioural profile and affected only the frequency of fish being stationary (not shown).

## 4 DISCUSSION

We tested drivers of sexual selection under clustered and dispersed substrate configurations in a substrate spawning fish, *N. furzeri*. We estimated male reproductive success, female mate choice and sex‐specific differences in behaviour. Males established a size‐dependent dominance hierarchy, and the largest, most dominant males formed clear territories and secured almost all matings when the spawning substrate was defendable (clustered). Females mated for a longer time with dominant males when spawning on the substrate. Hence, sexual selection clearly favours large males due to body size‐related dominance and female choice, apparently driving sexual dimorphism in this species. Behavioural differences between the sexes arose from male harassment of females and overall aggression, whereas females spent much more time feeding. Aggression was mainly intrasexual, irrespective of sex, suggesting the presence of intrasexual competition in both sexes. *N. furzeri* behaviour was dependent on time of day and water temperature, demonstrating their clear effect on behavioural scheduling, emphasizing the importance of controlling for these variables in behavioural studies (Nelson et al., 2024).

### 4.1 Dominance and territoriality

The largest male in the tub was always dominant. Dominance hierarchy was maintained by frequent (1.6 per minute) aggressive interactions with other males, which were initiated primarily by the large dominant males. This dominance was important for male reproductive success as dominant males secured ~80% matings when the spawning territory was defendable (i.e., clustered substrate), and they spawned twice as frequently at the preferred site (spawning substrate). A similar rate of dominant male success was observed in the laboratory experiments of Haas (1976) with *N. guentheri* (Pfeffer, 1893). Obtaining almost 80% of all matings is within the upper range of reproductive success of dominant males throughout the animal kingdom (Clutton‐Brock & Isvaran, 2006; Konečná et al., 2010) underlying the significance of the trait.

Dominant males formed territory around spawning substrate as they were observed in 55% of cases in the quadrant with substrate, when it was defendable. Reproductive territoriality is a common strategy in species with large males (Casalini et al., 2009; McCann, 1981). In *N. furzeri*, dominant male territoriality was motivated by substrate and not by female presence near the substrate, as females did not display a preference for the quadrant with the spawning substrate. Dispersed spawning substrate positions overwhelmed the dominant male's abilities to defend it, being non‐territorial, similar to other territorial species in such situations (Konečná et al., 2010). The strength of sexual selection, therefore, depends on how often spawning substrate is a limiting resource in the wild. Natural pools of *N. furzeri* vary in area as well as in the distribution of the highest‐quality substrate (Reichard, 2016). Overall, sexual selection in *Nothobranchius* killifishes clearly favours large males through their ability to monopolize spawning resources when they become scarce, a feasible scenario in spatially and temporally constrained ephemeral pools.

### 4.2 Aggression

*Nothobranchius furzeri* males spend more than half of their active time threatening or attacking conspecifics. Agonistic interactions were mostly ritualized non‐escalated attacks, with rare direct contact between the participants. Males competed over access to females, using aggression to maintain dominance. Correspondingly, aggression peaked in the afternoon when reproductive activity was the highest. The motivation for female–female aggression is less straightforward. We speculate that females may have competed over feeding opportunities as female–female aggression occurred mostly in the morning, when feeding was most intense. We do not suppose that females competed for spawning opportunities, as there was no association between female aggression and spawning frequency.

Males were also aggressive towards females. Intersexual aggression is frequently associated with female unresponsiveness to male spawning attempts (Garner et al., 2010). This is likely the case for *N. furzeri*, as males were the most aggressive towards females in the morning, when the frequency of spawning was the lowest. Male *N. furzeri* have been observed attacking unresponsive females in aquaria (Genade et al., 2005), sometimes leading to female mortality. This is likely related to coercive mating, common in live‐bearing relatives of the killifishes, such as eastern mosquitofish *Gambusia holbrooki* Girard, 1859, and guppies *Poecilia reticulata* Peters, 1859 (Bisazza et al., 2000; Magurran & Seghers, 1994).

### 4.3 Female mate choice

Females increased reproductive effort (by 40%) when two conditions were simultaneously met: (1) spawning with a dominant male and (2) spawning on the substrate. The female choice of the dominant male does not appear to confer any direct genetic benefit for embryo survival (Polačik & Reichard, 2009) but oviposition into the appropriate substrate might have direct implications for embryo survival in the wild, as the embryo will be exposed to harsh conditions of hypoxia and desiccation (Polačik et al., 2021). This concealed form of female mate choice is another factor contributing to the sexual size dimorphism in this species, as it further contributes to the reproductive success of large dominant males.

During our observations, *N. furzeri* males intensively pursued and harassed females. In fish species with external fertilization, coercive mating is less common (Matsumoto & Takegaki, 2016) but known in a few taxa forming temporary spawning pairs, such as salmonids or bleniids (Garner et al., 2010; Matsumoto & Takegaki, 2016; Watters, 2005). A previous laboratory study with *N. furzeri* reported that approximately half of the matings are coerced (Polačik & Reichard, 2011). Females may employ counter‐strategy to coercive mating, lowering the mating success of non‐preferred males by decreasing the number of laid eggs when mating has already begun (Reyer et al., 1999; Yamazaki & Koizumi, 2017) or purposely spawning on suboptimal substrate, which affects embryo survival (Bacon & Barbosa, 2020). In the present study, females reduced the number of spawning acts when mating with a subordinate male or outside the spawning substrate. This finding is in contrast with females of congeneric *Nothobranchius korthausae* Meinken, 1973 and *N. guentheri* (however, in a no‐choice experiment), which did not modulate the number of eggs laid in relation to male phenotype or dominance status (Haas, 1976; Polačik & Reichard, 2009). By conceding to coerced mating, females may lower the risk of injury (Genade et al., 2005) and decrease the net cost of interference with their foraging (Magurran & Seghers, 1994), which is directly linked to their fecundity (Vrtílek & Reichard, 2015). The cost of mating with a non‐preferred male in *N. furzeri* appears low, given that only a single egg is laid during each act, with up to 100 eggs produced daily (Vrtílek & Reichard, 2016). In addition, mating with non‐preferred males increases the genetic diversity of offspring (Reichard et al., 2007; Simmons, 2005), which may be favourable in an unpredictable environment of ephemeral pools because different (than current) phenotypes may be favoured during the next pool inundation. Overall, reproduction in *N. furzeri* is strongly shaped by male coercive mating but also includes an active female approach to mating.

### 4.4 Sex differences in behaviour

Sex‐specific evolutionary interests arising from contrasting selection pressures on each sex lead to behavioural differences (Fromhage & Jennions, 2016; Magurran & Maciás Garcia, 2000). Here, we show that the male time budget involves three times more agonistic interactions than for females, representing the most significant element of male locomotory behaviour. Previously, higher male than female activity was reported for *Nothobranchius* in both wild and semi‐natural conditions (Haas, 1976; Žák et al., 2019). Altogether, these extra energetic costs of males may slightly reduce reproductive cost differences between males and females, as suggested in other species (Šmejkal et al., 2017).

Females were feeding three times as frequently as males. The observed sex‐specific feeding frequency is not a consequence of male territoriality or dominance (p = 0.461, data not shown). Females are strongly motivated to feed to cover their energetic expenses for reproduction (Vrtílek & Reichard, 2016) including the consequences of intense male harassment. Previously, experimental shielding from male harassment and spawning considerably increased female somatic growth (Graf et al., 2010). Therefore, despite being half the body mass of males, females can eat a similar amount of food as males (Franck et al., 1998; Žák & Šuhajová, 2024).

The 10% higher participation of females in reproductive behaviour than for males may be due to observation bias, as only the behaviour of a single fish at a time was scored for the initial dataset, and the ID of other interacting fish was not recorded. In general, the striking differences in a behavioural repertoire between males and females were confirmed with different reproductive roles and dimorphism as probable causes.

## 5 CONCLUSION

Under the semi‐natural conditions of our study, with fish performing socially realistic interactions, we show that male–male competition is a major component of sexual selection in *N. furzeri* and leads to selection for male‐biased dimorphism. The dimorphism is maintained by the higher competitive ability and reproductive success of larger males. Spatial substrate configuration was a key for the expression of the male dominance hierarchy and territoriality. The spawning microhabitat, together with male dominance, increased female reproductive output. The behavioural patterns observed in *N. furzeri* are consistent with those reported in other egg‐laying Cyprinodontiforms (e.g., Haas, 1976; Kodric‐Brown, 1988). However, a comprehensive description of the natural behavioural repertoire for this species has been lacking, despite its relevance for biogerontologists and toxicologists studying treatment‐induced behavioural changes. This study provides a solid foundation for further examination of the behavioural ecology of *N. furzeri*, a remarkable species with increasing importance in various biological disciplines (Cellerino et al., 2016; Johnson & Jones, 2023; Reichard & Polačik, 2018; Thoré et al., 2021).

## AUTHOR CONTRIBUTIONS

Conceptualization: Jakub Žák and Martin Reichard; Methodology: Jakub Žák and Martin Reichard; Validation: Jakub Žák; Formal analysis: Jakub Žák; Investigation: Klára Mrkvová and Jakub Žák; Resources: Martin Reichard; Data curation: Jakub Žák; Writing – original draft: Jakub Žák and Martin Reichard; Writing – Review and editing: Martin Reichard, Jakub Žák and Klára Mrkvová; Visualization: Jakub Žák; Supervision: Martin Reichard and Jakub Žák; Funding acquisition: Martin Reichard.

## FUNDING INFORMATION

The study was funded by Czech Science Foundation (19‐01781S).

## CONFLICT OF INTEREST STATEMENT

The authors have no competing interests to declare that are relevant to the content of this article. Artificial intelligence has not been used to conceive, write and illustrate this report.

## Supporting information


**TABLE S1.** Table of tub‐specific male territoriality.
**TABLE S2.** Summary of the statistical approaches to the data analysis.
**TABLE S3.** Detailed statistical results of male dominance predictors.
**TABLE S4.** Detailed statistical results of the male dominance‐spawning probability relationship.
**TABLE S5.** ANOVA table of results for the dominance‐dependent place of spawning for males.
**TABLE S6.** Male territoriality model structure and the importance of predictors.
**TABLE S7.** Predictors of female spawning frequency (none significant).
**TABLE S8.** Statistical results for female mate choice, that is, length of spawning.
**TABLE S9.** Per‐minute frequency of all observed behaviours for each sex.
**TABLE S10.** Activity budget of *Nothobranchius furzeri*.
**FIGURE S1.** Day‐time‐dependent behaviour differences between *Nothobranchius furzeri* sexes.
**FIGURE S2.** Substrate‐dependent relationship of spawning probability in males with body mass.

**VIDEO S1.** Examples of behavioural interactions of the *Nothobranchius furzeri* in the mesocosm.

## Data Availability

The experimental data that support the findings of this study are available at FigShare repository 10.6084/m9.figshare.28025429 and 10.6084/m9.figshare.31011628.
