# Supplementary material for: Sexual selection and reproductive success in mesocosm populations of African annual killifish
Source: J Fish Biol. 2026 Mar 29;109(1):468–77. doi: 10.1111/jfb.70433 (PMC13397246; doi:10.1111/jfb.70433)
Supplement: Supplementary file 1 — TABLE S1. Table of tub‐specific male territoriality. TABLE S2. Summary of the statistical approaches to the data analysis. TABLE S3. Detailed statistical results of male dominance predictors. TABLE S4. Detailed statistical results of the male dominance‐spawning probability relationship. TABLE S5. ANOVA table of results for the dominance‐dependent place of spawning for males. TABLE S6. Male territoriality model structure and the importance of predictors. TABLE S7. Predictors of female spawning frequency (none significant). TABLE S8. Statistical results for female mate choice, that is, length of spawning. TABLE S9. Per‐minute frequency of all observed behaviours for each sex. TABLE S10. Activity budget of Nothobranchius furzeri. FIGURE S1. Day‐time‐dependent behaviour differences between Nothobranchius furzeri sexes. FIGURE S2. Substrate‐dependent relationship of spawning probability in males with body mass. [file JFB-109-468-s002.docx]

**SUPPORTING INFORMATION**

**Sexual selection and reproductive success in a mesocosm population of annual killifish**

Jakub Žák^1^*, Klára Mrkvová^1,2^, Martin Reichard^1,2,3^

1 Department of Botany and Zoology, Faculty of Science, Masaryk University, Kotlářská 2, Brno 611 37, Czech Republic

2 Institute of Vertebrate Biology, Czech Academy of Sciences, Květná 8, Brno 603 00, Czech Republic

3 Department of Ecology and Vertebrate Zoology, University of Lodz, Lodz, Poland

* Corresponding author: Jakub Žák, fish.jakub.zak@gmail.com

**ABSTRACT**

Variation in individuals’ ability to obtain mates generates sexual selection, which typically acts more strongly on males and can produce pronounced differences in phenotypes between the sexes (i.e., sexual dimorphism). The dynamic of sexual selection and individual reproductive success is considerably affected by the availability of reproductive territories. The *Nothobranchius furzeri* Jubb, 1971 (a model system in aging studies), inhabits temporally and spatially constrained ephemeral savanna pools where it lays its eggs in the muddy substrate of the pool. Males of the species are large and aggressive; nonetheless, it is unclear why it is dimorphic, whether they form territories, and how females select their partners for mating. We released 96 individuals into 12 semi-natural mesocosms with either a clustered or a dispersed configuration of spawning substrate. We found that males formed body size-dependent dominance hierarchies. Dominant males were territorial and nearly monopolised matings when the substrate was defendable. Females engaged in 40% more spawning acts within a spawning bout with dominant males. Therefore, the intersexual size dimorphism appears to be maintained by the higher competitive ability and reproductive success of large males. Females were less aggressive, but harassed by males, and fed three times more than males. Overall, we demonstrated under a semi-natural setting that the mating system in *N. furzeri* is dominated by large males, which become territorial when the spawning substrate is limited (clustered), a likely situation in the environment of ephemeral pools.

_______________

Video of the behavioural interactions: https://doi.org/10.6084/m9.figshare.28025453

The dataset: 10.6084/m9.figshare.28025429

The Additional Supporting Information: https://doi.org/10.6084/m9.figshare.28025429


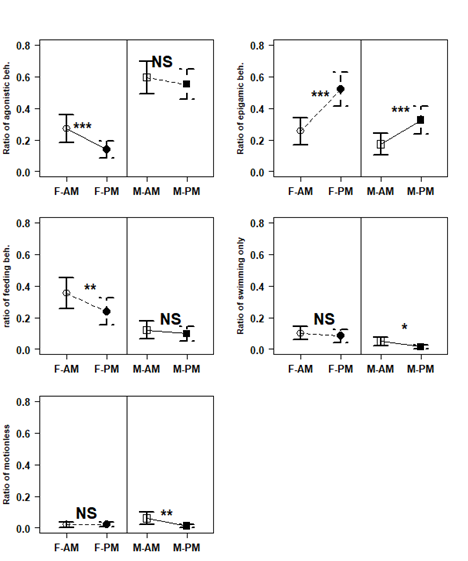
**SUPPORTING FIGURES**

SUPPORTING FIGURE S1: TEMPORAL ASPECT OF SEX-SPECIFIC BEHAVIOUR

**Supporting Figure S1 Legend**: Sex and day period specific ratio of behaviours in the mesocosm population of *Nothobranchius furzeri* (N=1,895). Points are mean model estimated (BCMM) ratios of a specific behaviour in relation to other behaviours (i.e., sum of y-axis of behaviours is 1). Circle points are for females, and square points are for males. Empty points are for morning. Filled points are for the afternoon. Error bars indicate 95% confidence intervals. F-AM: relative ratio of a specific behaviour to other behaviours observed for females in the morning. Morning was considered as time period of 7am to 2pm. M-PM: ratio of a specific behaviour for males in the afternoon. The afternoon was considered a time period from 2 pm to 8pm. NS – non significant, * - p value 0.011 – 0.050, ** - p value 0.01 – 0.0011, *** - p value <0.0011. Pairwise within “time-of-the-day” contrasts were computed in the emmeans package (Lenth, 2023).

Behavioural repertoire of each sex differed between morning and evening (BCMM, *sex: time-of-the-day* interaction, p <0.001). While males were involved in agonistic behaviour throughout the day (z = 0.82, p = 0.410), females were involved in agonistic behaviour twice as frequently in the morning than in the afternoon (z = 3.10, p = 0.002). Reproductive behaviour was more frequent in the afternoon in both sexes (females: z = 5.25, p < 0.001, males: z = 3.41, p = 0.001). Feeding was more common in the morning than in the afternoon in females (z = 2.63, p = 0.008) but not in males (z = 0.94, p = 0.348). Males were recorded as stationary (z = 2.60, p = 0.009) and swimming (z = 2.42, p = 0.0157) more frequently in the morning than in the afternoon. In contrast, these behaviors were recorded at a similar rate throughout the day for females (p = 0.508 – 0.886). Concordant results were obtained after removing agonistic behaviour from the analysis.

There was also significant *sex:substrate configuration* interaction, which only affected the number of motionless fish, and not the other behaviours.

SUPPORTING FIGURE S2: Substrate-distribution dependent relationship of male body mass with male spawning probability in mesocosm population of *Nothobranchius furzeri*


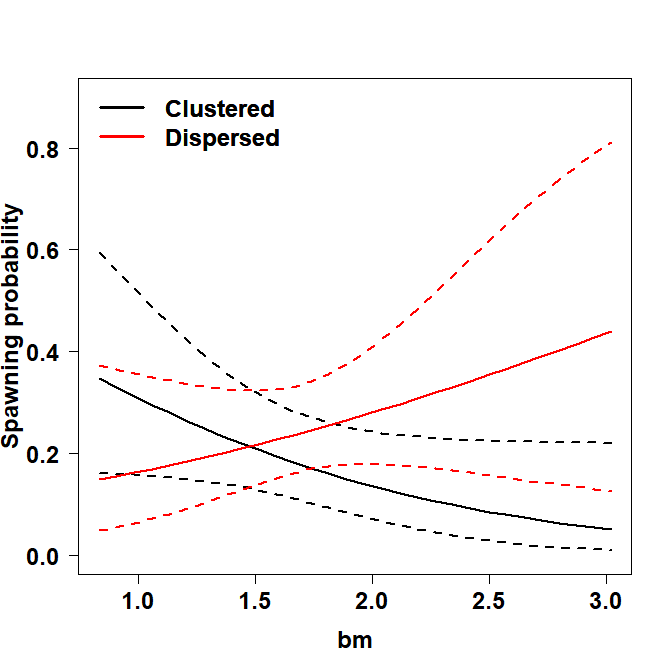


**Supporting Figure S2** Substrate-distribution-dependent relationship of male body mass with male spawning probability in mesocosm population of *Nothobranchius furzeri*. Results of a Binomial GLMM model showed a significant (p=0.044) male *body-mass:substrate distribution* interaction. Chances of male spawning are positively related to male body mass when the spawning substrate was dispersed. In contrast, spawning chances were more dominance-dependent when substrate was clustered (see Results and Fig.2b). Dashed lines represent 95% CI. bm – on x-axis stands for body mass in grams

**SUPPORTING TABLES**

SUPPORTING TABLE S1: TUB-SPECIFIC MALE TERITORIALITY

**Supporting Table S1** The raw ratio of an occurrence of dominant *Nothobranchius furzeri* male in the respective quadrant of the mesocosm. Quadrant 1 represents a quadrant where substrate was clustered in a treatment with clustered substrate. Red cells indicate significantly more frequent presence of a dominant male in a respective quadrant. There is a preferred quadrant in 5 out of 6 tubs with clustered substrate and only one in the tub with dispersed substrate. “D” next to tub number represents dispersed substrate in tub while “C” represents clustered substrate (grey rows). (N of records for clustered substrate = 405; N of records for dispersed substrate = 484). For clustered substrate, this is an underestimate of the overall time spent in the territory, as dominant males frequently briefly visited several quadrants (which diluted the importance of quadrant with substrate in the multinomial model) to pursue males or females within the observation period rather than spending more time in that part of the habitat. When we would use just the binomial recording of whether male was recorded in quadrant with substrate or not, the proportion of presence data (territoriality) would be much (~20 %) higher than the applied multinomial approach. It should be noted that male territoriality was stable over the day and over the entire (almost one-month-long) observation period.

| Tub | Quadrant 1 | Quadrant 2 | Quadrant 3 | Quadrant 4 |
| --- | --- | --- | --- | --- |
| 1D | 0.13 | 0.20 | **0.40** | 0.27 |
| 2C | 0.29 | 0.12 | 0.29 | 0.29 |
| 3D | 0.25 | 0.31 | 0.13 | 0.31 |
| 4D | 0.27 | 0.31 | 0.23 | 0.19 |
| 5D | 0.23 | 0.31 | 0.31 | 0.15 |
| 6C | **0.69** | 0.00 | 0.15 | 0.15 |
| 7D | 0.29 | 0.18 | 0.24 | 0.29 |
| 8C | **0.64** | 0.09 | 0.18 | 0.09 |
| 9C | **0.67** | 0.07 | 0.07 | 0.20 |
| 10C | **0.73** | 0.00 | 0.18 | 0.09 |
| 11C | **0.47** | 0.12 | 0.18 | 0.24 |
| 12D | 0.25 | 0.29 | 0.25 | 0.21 |

|  | Trait of Interest | Analytical Approach | Output can be Found at |
| --- | --- | --- | --- |
| 1 | Male Dominance | GLMM (Binomial distribution) | Fig 2a, Supporting Table S3 |
| 2 | Male Hierarchy Dependent Spawning Probability | GLMM (Binomial distribution) | Fig. 2b,  Supporting Table S4, Supporting Figure S5 |
| 3 | Spawning into/outside Spawning Substrate in Relation to Male Hierarchy | GLMM (Binomial distribution) | Fig. 2c, Supporting Table S5 |
| 4 | Male Hierarchy Dependent Territoriality | BCMM | Supporting Table S1  Supporting Table S6 |
| 5 | Quadrant-Specific Distribution of Females | BCMM | Text in results only |
| 6 | Sex-Specific Direction of Agonistic Interactions | BCMM | Fig 3b |
| 7 | Structure of Reproductive Behaviour | BCMM | Fig 3c |
| 8 | Sex-Specific Behavioural Profile (activity budgets) | BCMM | Fig 3a, Supporting Figure S1 Supporting Table S9 Supporting Table S10 |
| 9 | Probability of Spawning in Females | GLMM (Binomial distribution) | Supporting Table S7 |
| 10 | Number of Female Spawning Acts in Relation to Partner/Male Dominance | GLMM (Gamma distribution) | Fig. 2d, Supporting Table S8 |

SUPPORTING TABLE S2: Overview of the analyzed traits, the approaches employed for their statistical analysis and where the outputs are presented within the paper. All models were fitted with all possible two-way interactions. The most parsimonious models were chosen by backward selection by removing non-significant (p > 0.05) interactions. The predictor significance of BCMM models was estimated by Analysis of Deviance comparison of models with and without the predictor/interaction of interest.

_______________________________________________________________________

SUPPORTING TABLE S3: PREDICTORS OF DOMINANCE IN MALES

**Supporting Table S3:** ANOVA(type III) table of the GLMM model with binomial error distribution describing the predictors of male dominance. The response variable is dominance, where each agonistic interaction observed for each male was recorded as “1” if the male was the initiator of the agonistic interaction, and “0” if the male was the recipient. (Nrecords = 631, Nobservations =255, Nmales=48, Ntubs =12)

Full model:

glmer(dominance~(bm+sub.distribution+halfday+temperature)^2+(1|tub/id)+(1|observation),binomial

| Predictor | χ2 (df) | Pr(>Chisq) |
| --- | --- | --- |
| Intercept | **7.87 (1)** | **0.005** |
| Body mass | **19.88 (1)** | **<0.001** |
| Substrate distribution | 0.06 (1) | 0.792 |
| Time of the day | 0.94 (1) | 0.329 |
| Water temperature | 0.37 (1) | 0.539 |

_______________________________________________________________________

SUPPORTING TABLE S4: DOMINANCE RELATED SPAWNING PROBABILITY IN MALES

**Supporting Table S4** Probability of a male being observed spawning in relation to its dominance and spawning substrate distribution. Values shown are only for fixed factors. The results of the most parsimonious model of Binomial GLMM (ANOVA table type III). *Male ID* nested within *Tub ID* was a nested random factor and *ID of the observation interval* was simple random factor. The full model contained all possible double interactions. N = 363, N_tubs_ =12, N_observation intervals_ = 91, N_males_ =48

| predictor | χ2 (df) | Pr(>Chisq) |
| --- | --- | --- |
| **intercept** | **5.09 (1)** | **0.024** |
| **Dominance** | **25.18 (3)** | **<0.001** |
| Body mass | 3.66 (1) | 0.056 |
| **Substrate** | **6.12 (1)** | **0.014** |
| **Dominance:substrate** | **12.74(3)** | **0.005** |
| **Body mass:substrate** | **4.45(1)** | **0.035** |

_______________________________________________________________________

SUPPORTING TABLE S5: PROBABILITY OF MALES SPAWNING INTO THE SPAWNING SUBSTRATE

**Supporting Table S5** Anova table (type III) of the model analyzing the probability of *Nothobranchius furzeri* male spawning into the spawning substrate in relation to male dominance and substrate distribution within the mesocosm experiment. The results of the Generalized linear mixed effect model with binomial error distribution. The tesponse variable was a proportion computed from the *sum of the spawning frequency (number of spawnings per minute) into the spawning substrate* vs. *sum of frequency of spawning outside* the spawning substrate. The male dominance factor had two levels: 1/ the most dominant male, 2/other subordinate males (sub). N=39 (there were no spawning records for 9 males, so they were not included in the analysis)

| Predictor | χ2 (df) | Pr(>Chisq) |
| --- | --- | --- |
| Intercept | 0.05(1) | 0.823 |
| Male dominance | **4.23**(1) | **0.039** |
| Substrate distribution | 0.07(1) | 0.794 |
| Male body mass | 0.13(1) | 0.720 |

___________________________________________________________________________

SUPPORTING TABLE S6: MALE TERITORIALITY

**Supporting Table S6**: Importance of predictors in the model investigating the relationship of male dominance with quadrant preference (i.e., territoriality). Full model contained all possible double interactions of the following predictors: *male dominance* category, *time-of-the-day* and *water temperature*. *Male ID* (N=24) nested within *tub ID* (N=6) were nested random factors, and the *ID of observation interval* (N=189) was a simple random factor. Results are based on 405 records of male positions in tubs. P-values come from the Analysis of deviance from model comparisons between the full model and a model without the variable of interest. P-values for full model and model without interactions are comparisons with model with intercept only (first 3 rows of the table). P-values for simple predictors are comparisons with model without interactions. P-values for interactions are from comparisons with a model including all double interactions (bottom three rows)

| Predictor | AIC(df) | p |
| --- | --- | --- |
| ~1 + (1\|TubID/Male ID)+(1\|Interval) | 1164 (21) | NA |
| Full model with all double interactions | 1207 (57) | 0.783 |
| Model without interactions | 1181 (36) | 0.580 |
| Predictors from the model with interactions |  |  |
| Male dominance | 1167(27) | 0.919 |
| time-of-the-day | 1178(33) | 0.378 |
| Water temperature | 1178(33) | 0.458 |
| Male dominance: time-of-the-day | 1200(48) | 0.332 |
| Male dominance: water temperature | 1200(48) | 0.281 |
| time-of-the-day: water temperature | 1202(54) | 0.829 |

_____________________________________________________________________

SUPPORTING TABLE S7: FEMALE SPAWNING PROBABILITY

**Supporting Table S7** Results of the full model, including double interactions (Binomial GLMM) predicting female spawning probability (within 5-minute observ. interval) in *Nothobranchius furzeri* from the mesocosm experiment. Results are based on 376 records from 91 observation intervals. (N_females_=48, N_tubs_ = 12)

| Predictor | χ2(df) | p |  |
| --- | --- | --- | --- |
| Dominance category of female | 0.612(3) | 0.894 |  |
| Body mass | 0.221(1) | 0.638 |  |
| Substrate distribution | 0.048(1) | 0.828 |  |
| Dominance cat.:body mass | 2.026(3) | 0.567 |  |
| Dominance cat.: substrate dist. | 1.149(3) | 0.765 |  |
| Body mass:substrate dist. | 0.089(1) | 0.766 |  |

___________________________________________________________________________

SUPPORTING TABLE S8: FEMALE MATE CHOICE

**Supporting Table S****8** Number of spawning acts within a reproductive bout (N=185) with male in relation to male dominance and place of spawning (inside/outside) substrate. Results of Gamma GLMM. All double interactions were removed because they were insignificant (p>0.25). leaving only significant *male dominance:place of spawning* interaction. N_tubs_ = 12 (2 omitted without spawnings), N _Observation ID_ = 72, N_females_ =41 (7 omitted without spawning). The model structure was:

Full model:

Number of spw bouts~(male dominance+female bm+substrate distribution+place of spawning)^2+(1|tub ID/IDF)+(1|observation ID)

| Predictor | χ2 (df) | P |
| --- | --- | --- |
| **Male dominance** | **11.348 (1)** | **<0.001** |
| Female body mass | 0.114(1) | 0.736 |
| Substrate distribution | 0.455(1) | 0.500 |
| **Place of spawning** | **14.101(1)** | **<0.001** |
| **Male dominance: place of spawning** | **7.998(1)** | **0.004** |

___________________________________________________________________________

SUPPORTING TABLE S9: FREQUENCY OF OBSERVED BEHAVIOURS PER MINUTE IN MESOCOSM POPULATION OF *NOTHOBRANCHIUS FURZERI*

**Supporting Table S9** Raw means of frequency of observed behaviours per one minute of recording of the mesocosm population of *Nothobranchius furzeri*. Values are extracted from the raw dataset, uncorrected for treatment (substrate distribution), time-of-day and water temperature or animal body size. SD – standard deviation

| Behaviour | Female \|#N per min (SD) | Male \|#N per min (SD) |
| --- | --- | --- |
| Agonistic | 0.45 (0.862) | 1.65 (1.650) |
| Reproductive | 0.72 (1.061) | 0.68 (1.122) |
| Feeding | 0.68 (1.369) | 0.33 (0.878) |
| Swimming only | 0.23 (0.422) | 0.11 (0.313) |
| Stationary | 0.05 (0.217) | 0.08 (0.273) |

SUPPORTING TABLE S10: SEX-SPECIFIC ACTIVITY BUDGETS (BEHAVIOURAL PROFILES)

**Supporting Table S10** Sex-specific activity budget of mesocosm population of *Nothobranchius furzeri*. Each value represents the percentage of time each sex was involved (both initiator and recipient) in a specific behaviour. Values are averaged over effects of daytime, substrate distribution and temperature. Values in brackets indicate percentages for specific category of behaviour (e.g. 30 (78) for “female chasing” means that 30 % of all recorded behavivours for females represent chasing and 77.5 % of recorded reproductive (epigamic) behaviours represent chasing in females). Values in brackets for the agonistic behaviour are making 100 when summarized over intersex and intrasex values for males and females together. Lateral display in reproductive behaviour is only reported from raw percentages and not as model estimates, because it was removed from the analysis due to small sample size (3 received females, 4 showed by males). Therefore, the sum of reproductive behaviour is slightly above 100. Lateral display, chasing, contact and spawning are all subcategories of the reproductive (epigamic) behaviour

|  | FEMALE  Raw activity budget  (%) | FEMALE  Detailed activity budget  (%) |  | MALE Raw activity budget (%) | MALE Detailed activity budget (%) |  |
| --- | --- | --- | --- | --- | --- | --- |
| Agonistic | 20 | - | - | 57 | - | - |
| Intrasex agonistic | - | 19.8 | (11.5) | - | 46.4 | (72.3) |
| Intersex agonistic | - | 0.2 | (0.1) | - | 10.6 | (16.5) |
| Reproductive | 39 | - | - | 25 | - | - |
| Lateral display | - | - | (0.1) | - | - | (0.2) |
| Chasing | - | 30 | (77.5) | - | 17 | (66.5) |
| Contact | - | 6 | (15.7) | - | 5 | (21.5) |
| Spawning | - | 3 | (6.8) | - | 3 | (12.0) |
| Feeding | 30 | 30 | - | 11 | 11 | - |
| Swimming only | 9 | 9 | - | 3 | 3 | - |
| Stationary | 2 | 2 | - | 4 | 4 | - |
| TOTAL | 100 | 100 | - | 100 | 100 | - |

REFERENCES CITED IN THE SUPPORTING

Reichard, M. (2016). The evolutionary ecology of african annual fishes. In N. Berois, G. Garcia, & R. O. De Sá (Eds.), *Annual Fishes; Life History Strategy, Diversity, and Evolution* (pp. 133–158). CRC Press.

Smith, P., Willemsen, D., Popkes, M., Metge, F., Gandiwa, E., Reichard, M., & Riccardo Valenzano, D. R. (2017). Regulation of life span by the gut microbiota in the short-lived African turquoise killifish. *ELife*, *6*, e27014. https://doi.org/10.7554/eLife.27014.001

Žák, J., Reichard, M., & Gvoždík, L. (2018). Limited differentiation of fundamental thermal niches within the killifish assemblage from shallow temporary waters. *Journal of Thermal Biology*, *78*, 257–262. https://doi.org/10.1016/j.jtherbio.2018.10.015
